# *cacna2d3*, a voltage-gated calcium channel subunit, functions in vertebrate habituation learning and the startle sensitivity threshold

**DOI:** 10.1371/journal.pone.0270903

**Published:** 2022-07-14

**Authors:** Nicholas J. Santistevan, Jessica C. Nelson, Elelbin A. Ortiz, Andrew H. Miller, Dima Kenj Halabi, Zoë A. Sippl, Michael Granato, Yevgenya Grinblat

**Affiliations:** 1 Department of Integrative Biology, University of Wisconsin, Madison, Wisconsin, United States of America; 2 Department of Neuroscience, University of Wisconsin, Madison, Wisconsin, United States of America; 3 Genetics Ph.D. Training Program, University of Wisconsin, Madison, Wisconsin, United States of America; 4 Department of Cell and Developmental Biology, University of Pennsylvania, Philadelphia, PA, United States of America; 5 Neuroscience Graduate Program, University of Pennsylvania, Philadelphia, PA, United States of America; 6 Neuroscience Ph.D. Training Program, University of Wisconsin, Madison, Wisconsin, United States of America; Indiana University School of Medicine, UNITED STATES

## Abstract

**Background:**

The ability to filter sensory information into relevant versus irrelevant stimuli is a fundamental, conserved property of the central nervous system and is accomplished in part through habituation learning. Synaptic plasticity that underlies habituation learning has been described at the cellular level, yet the genetic regulators of this plasticity remain poorly understood, as do circuits that mediate sensory filtering.

**Methods:**

To identify genes critical for plasticity, a forward genetic screen for zebrafish genes that mediate habituation learning was performed, which identified a mutant allele, *dory*^*p177*^, that caused reduced habituation of the acoustic startle response. In this study, we combine whole-genome sequencing with behavioral analyses to characterize and identify the gene affected in *dory*^*p177*^ mutants.

**Results:**

Whole-genome sequencing identified the *calcium voltage-gated channel auxiliary subunit alpha-2/delta-*3 (*cacna2d3*) as a candidate gene affected in *dory*^*p177*^ mutants. Behavioral characterization of larvae homozygous for two additional, independently derived mutant alleles of *cacna2d3*, together with failure of these alleles to complement *dory*^*p177*^, confirmed a critical role for *cacna2d3* in habituation learning. Notably, detailed analyses of the acoustic response in mutant larvae also revealed increased startle sensitivity to acoustic stimuli, suggesting a broader role for *cacna2d3* in controlling innate response thresholds to acoustic stimuli.

**Conclusions:**

Taken together, our data demonstrate a critical role for *cacna2d3* in sensory filtering, a process that is disrupted in human CNS disorders, e.g. ADHD, schizophrenia, and autism.

## Introduction

To successfully navigate their environments, animals continuously adjust their behaviors to ensure that they are appropriate for the current environment. Their nervous systems must quickly process incoming stimuli to distinguish relevant from irrelevant information, which allows for focused attention and supports higher executive functions like memory formation and behavioral regulation. Sensory filtering is mediated in part by the fundamental and conserved process of habituation [1]. Habituation, the simplest form of non-associative learning exhibited by all animals, is defined as a progressive decline in responsiveness to repeated, insignificant stimuli [2], and is not due to sensory adaptation or motor fatigue [3]. Notably, it has been shown that animals are also able to habituate to threatening and potentially lethal stimuli as a means of modifying their behavioral strategy to avoid dangerous stimuli [4]. The behavioral parameters and cellular mechanisms of habituation are controlled by synaptic plasticity mechanisms that alter neurotransmitter signaling to regulate a balance of excitation and inhibition [5–9], but our knowledge of the critical genes that mediate habituation is incomplete.

Impairment of filtering mechanisms is a hallmark of many common neurological disorders, so much so that habituation deficits have been used as a diagnostic tool [10]. Habituation deficits are associated with autism spectrum disorders (ASD) [11–13], Fragile X syndrome [14], schizophrenia [15], Huntington’s disease [16], attention deficit hyperactivity disorder (ADHD) [17], Parkinson’s disease [18], Tourette’s syndrome [19], and migraine [20]. Dissecting the underlying genetic mechanisms that regulate sensory filtering can provide insight into the etiology of disease, identify genetic predispositions for diseases, and identify potential therapeutic targets. Importantly, understanding the genetic, cellular, and behavioral aspects of habituation is critical to understanding how normal neural circuits process sensory information.

Zebrafish can perform sensory-evoked motor behaviors that are modulated by experience by 5 days post-fertilization (dpf). Acoustic stimuli elicit one of two distinct motor responses in zebrafish: a short-latency C-bend (SLC), generally performed in response to a high-intensity stimulus, and a long-latency C-bend (LLC), generally performed in response to a low-intensity stimulus [21]. These behaviors are driven by simple, well-characterized circuits that are accessible to visualization and genetic manipulation [22]. A SLC is triggered by activating one of two bilateral Mauthner hindbrain reticulospinal neurons, the command neurons of the acoustic startle response (ASR) [23]. The Mauthner neuron is functionally analogous to the giant neurons of the caudal pontine reticular nucleus (PnC), which receive input from the cochlear nerve and output to motor neurons in the spinal cord to drive the startle response in mammals [24–26]. While the zebrafish circuitry is simpler in comparison to that of mammals, it is this simplicity that makes it a useful tool for investigating the genetic, cellular and behavior mechanisms that underlie sensory filtering.

To identify genes that are important for mediating habituation learning, we combined a genome-wide forward genetic screen [27] with a high-throughput platform for unbiased acoustic startle analysis [28]. This approach yielded several genes required for acoustic startle habituation, including the palmitoyltransferase *Huntingtin interacting protein 14 (hip14)* [29], the *adaptor related protein complex 2 subunit sigma 1* gene (*ap2s1*) [30], the extracellular metalloprotease *pregnancy associated plasma protein-aa (pappaa)*, and the enzyme *pyruvate carboxylase a (pcxa)* [27]. Here, we report that a previously identified mutant allele [27], named *dory*^*p177*^, is a mutation in the *calcium voltage-gated channel auxiliary subunit alpha-2/delta-3* (*cacna2d3*) gene, predicted to result in a premature stop codon. *cacna2d3* encodes a member of the α2δ subunit family of proteins in the voltage-gated calcium channel (VGCC) complex, known to be involved in synaptic transmission and neurotransmitter release [31,32]. We show that *cacna2d3* is required for vertebrate sensory filtering, with *cacna2d3* mutant zebrafish exhibiting reduced habituation and a reduced innate startle threshold to acoustic stimuli. Collectively, these data show that *cacna2d3* plays an important role in sensory filtering by controlling both the innate acoustic startle threshold and the ability of the animal to habituate to repeated, irrelevant acoustic stimuli.

## Results

### Whole-genome sequencing identifies cacna2d3 as a candidate regulator of habituation learning

**Fig 1:**
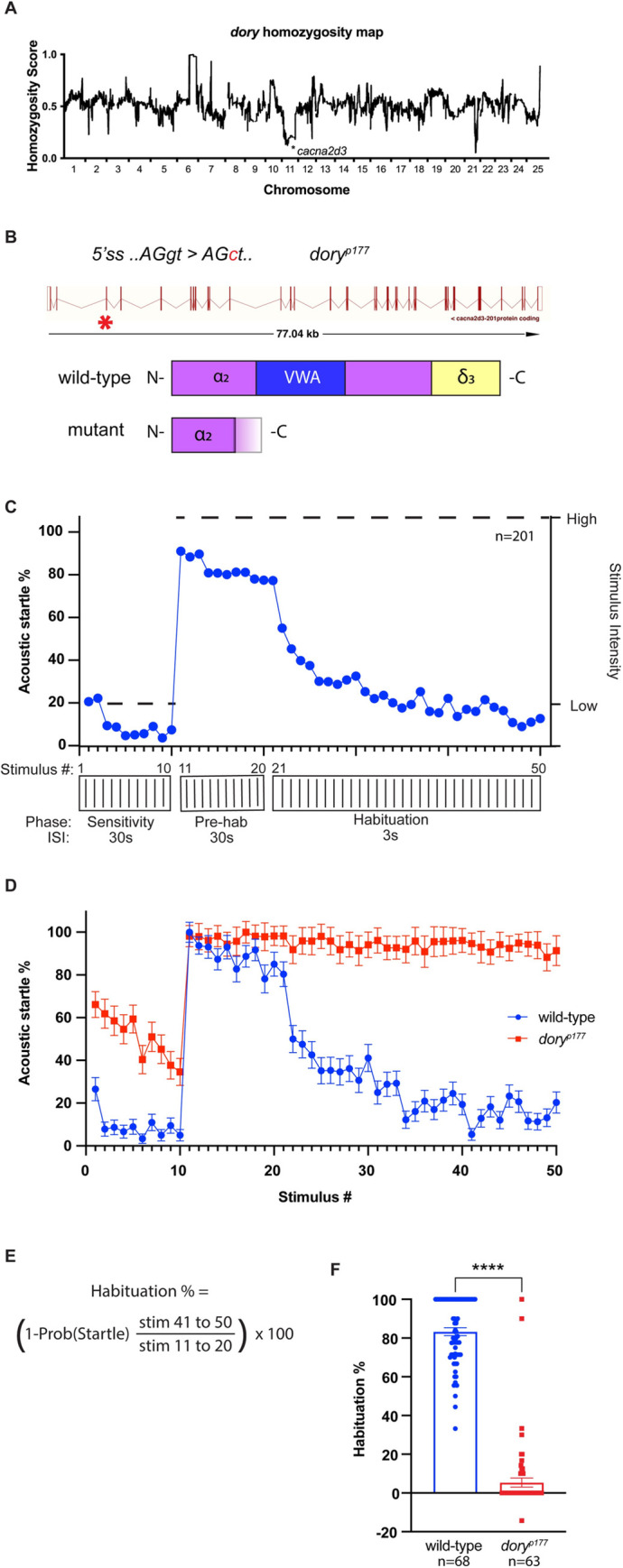
*dory*^*p177*^, linked to cacna2d3 by homozygosity analysis, causes reduced habituation of the acoustic startle response. (A) Homozygosity plot of *dory*^*p177*^ mutants based on whole genome sequencing results. Homozygosity scores close to 1.0 indicate linkage to TL alleles while scores close to 0.0 indicate linkage to WIK alleles. Asterisk indicates the position of the identified splice-donor site mutation in *cacna2d3* on chromosome 11. (B) Schematics of the *cacna2d3* gene and the amino acid sequences encoded by the wild-type *cacna2d3* allele and the *dory*^*p177*^ mutant allele. In the *cacna2d3* gene diagram, the site of the point mutation in *dory*^*p177*^ mutants is indicated by a red asterisk. (C) Schematic representation of the acoustic startle habituation assay. Larvae were exposed to 10 “sub-threshold” low-intensity acoustic stimuli delivered at 30s interstimulus intervals (ISI) to access startle sensitivity. Next larvae were exposed to 10 high-intensity non-habituating stimuli delivered at 30s ISI to determine baseline startle responsiveness followed by 30 high intensity habituating stimuli at a 3s ISI to access habituation. The blue plot was generated by pooling control animal responses to demonstrate a typical wild-type response at each phase of the assay. (D) Mean acoustic startle responsiveness of wild-type (shown in blue) and *dory*^*p177*^ mutants (shown in red) to each of the stimuli presented during the habituation assay. (E) Mean acoustic startle habituation percentage is calculated by taking the ratio of the mean frequency of startle responsiveness (startle probability) of each larva to stimuli 41–50 over stimuli 11–20. (F) Mean acoustic startle habituation percentage of wild-type and *dory*^*p177*^ mutants. Wild-type larvae are shown in blue and homozygous *dory*^*p177*^ mutants are shown in red. Number of larvae shown below each bar. ****p<0.0001, Mann-Whitney test versus wild-type. Error bars indicate SEM.

Habituation is defined as a progressive decline in responsiveness to repeated, insignificant stimuli [2]. Habituation is the simplest form of non-associative learning found in all animals, yet its genetic regulation remains poorly understood. To identify genes involved in habituation, we combined forward genetic mutagenesis screening with a robust, unbiased behavioral assay for acoustic startle habituation [27]. *dory*^*p177*^ was among the mutant allele collection generated through this approach. To identify the likely causative mutation underlying the *dory*^*p177*^ habituation defect, whole genome sequencing (WGS) was performed followed by homozygosity mapping. We mapped *dory*^*p177*^ to a chromosome 11 (Fig 1A) interval containing a unique mutation in the *calcium voltage-gated channel auxiliary subunit alpha-2/delta-3* gene, *cacna2d3* (Fig 1B).

Cacna2d3 is a member of the α2δ subunit family of proteins, which regulate the trafficking and surface expression of VGCC complexes [33]; VGCCs in turn modulate synaptic transmission and function [32,34,35]. The primary sequence of α2δ3 is strongly conserved across vertebrates; human CACNA2D3 shares 76.5% amino acid identity with zebrafish Cacna2d3 protein, 87.6% similarity along the length of the protein, and 89.4% amino acid identity/93.4% similarity in the Von Willebrand Factor A (VWA) functional domain (S1 Fig). The unique thymine to cytosine single base pair substitution in the *dory*^*p177*^ allele resides at the donor splice site of *cacna2d3* intron 2–3. This mutation is predicted to either cause a partial retention of the intronic sequence, cause skipping of exon 2, or activate a cryptic donor site. The first two scenarios (S2 Fig) lead to frameshifts and generation of mutant proteins lacking the VWA domain and the *δ-3* subunit that anchors Cacna2d3 to the cell membrane (schematized in Fig 1B), both critical to Cacna2d3 function in VGCCs [32].

### *cacna2d3* regulates habituation learning

We measured the ASR of *dory*^*p177*^ mutant larvae versus wild-type (TL) larvae using an automated behavioral platform that delivers acoustic stimuli of defined intensity, records behavioral responses with a high-speed camera, and tracks each animal’s movement to evaluate the initiation and kinematic performance of ASR behavior [21,28]. Larvae were exposed to a series of 50 acoustic stimuli (Fig 1C). The first 10 stimuli (the “sensitivity” phase) were delivered at a subthreshold low-level intensity and spaced at 30-second intervals to assess startle sensitivity. The intensity of these subthreshold weak stimuli was chosen empirically to elicit SLCs ~10% of the time in wild-type larvae during this phase. The next 10 stimuli (the “pre-habituation” phase) were delivered at a high-level intensity and spaced at non-habituating intervals of 30 seconds to determine baseline acoustic startle responsiveness. The intensity of these strong stimuli was set empirically to elicit SLCs ~80% of the time in wild-type larvae during this phase. The following 30 stimuli (the “habituation” phase) were delivered at the same high-level intensity, but spaced only 3 seconds apart, which elicits short-term habituation. The mean acoustic startle responsiveness of wild-type and *dory*^*p177*^ mutants to each stimulus is shown in Fig 1D. Habituation is calculated as a fraction of the final mean acoustic startle responsiveness (stimuli 41–50) over the initial mean responsiveness (stimuli 11–20) (Fig 1E). Under these conditions, wild-type (TL) larvae show a rapid reduction in SLC startle frequency and stereotypically habituate by more than 80% (Fig 1F). In this assay, *dory*^*p177*^ homozygotes exhibited startle habituation of only 5.4% compared to the wild-type control larvae, which habituated by 83.3% (Fig 1F; wild-type controls n = 68, *dory* ^*p177*^ homozygotes n = 63).

**Fig 2:**
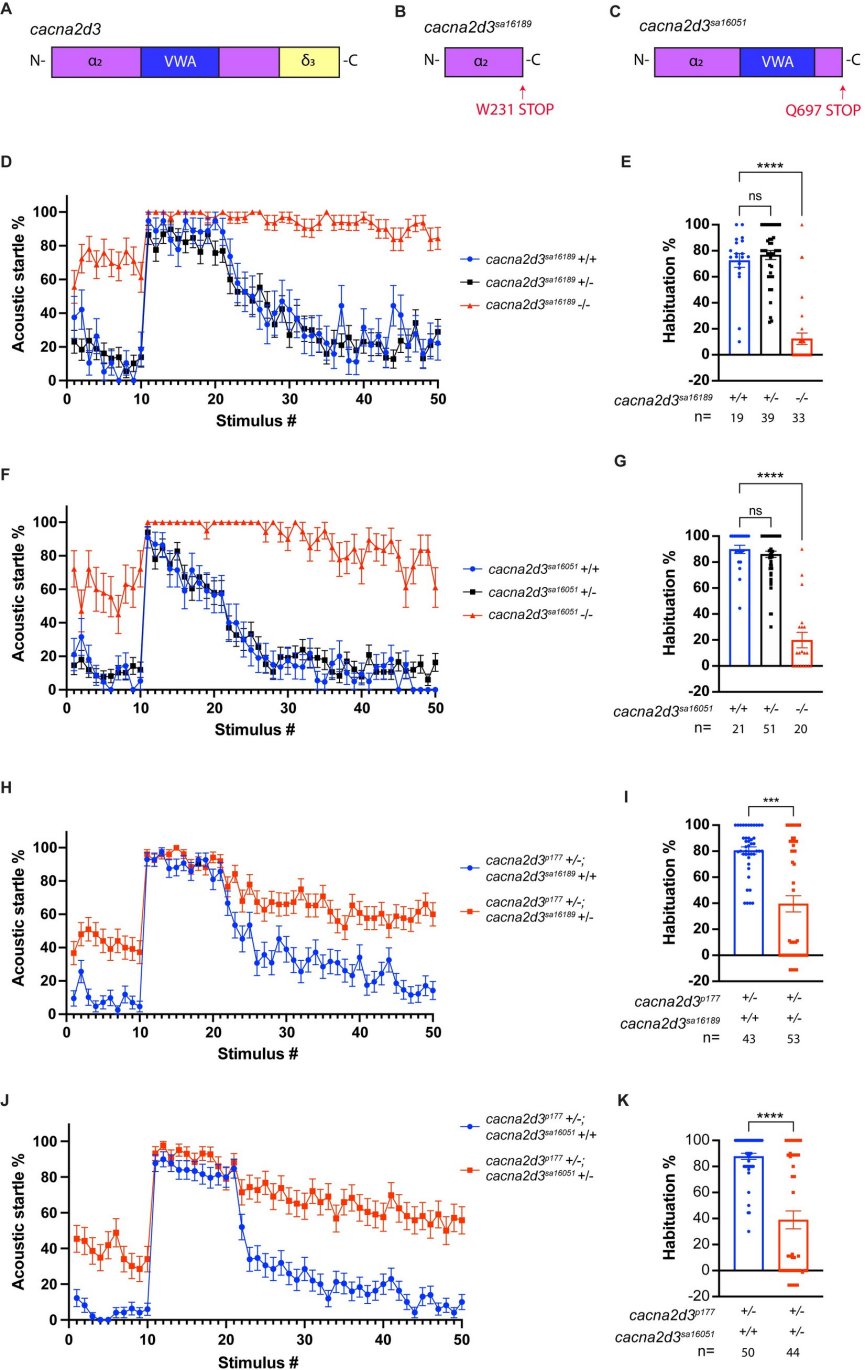
*cacna2d3* mutants and trans-heterozygotes exhibit reduced ASR habituation. (A-C) Schematics of the wild-type *cacna2d3* allele (A), the *cacna2d3*^*sa16189*^ mutant allele (B), and the *cacna2d3*^*sa16051*^ mutant allele (C). (D) Mean acoustic startle responsiveness to each stimulus in the habituation assay. Animals were generated from a *cacna2d3*^*sa16189*^*/+* incross. Wild-type larvae are shown in blue, *cacna2d3*^*sa16189*^*/+* heterozygotes are shown in black, and homozygous *cacna2d3*^*sa16189*^ mutants are shown in red. (E) Mean acoustic startle habituation percentage. (F) Mean acoustic startle responsiveness to each stimulus of the habituation assay. Animals were generated from a *cacna2d3*^*sa16051*^*/+* incross. Wild-type larvae are shown in blue, *cacna2d3*^*sa16051*^*/+* heterozygotes are shown in black, and homozygous *cacna2d3*^*sa16051*^ mutants are shown in red. (G) Mean acoustic startle habituation percentage. (E, G) ***p<0.001, ****p<0.0001, one-way ANOVA with Dunnett’s multiple comparison test. (H) Mean acoustic startle responsiveness to each stimulus of the habituation assay. Animals were generated from a cross between *cacna2d3*^*p177*^ mutants with *cacna2d3*^*sa16189*^*/+* heterozygotes. *cacna2d3*^*p177*^*/*+ larvae are shown in blue and *cacna2d3*^*p177*^/*cacna2d3*^*sa16189*^ trans-heterozygotes are shown in red. (I) Mean acoustic startle habituation percentage. (J) Mean acoustic startle responsiveness to each stimulus of the habituation assay. Animals were generated from a cross between *cacna2d3*^*p177*^ mutants and *cacna2d3*^*sa16051*^*/+* heterozygotes. *cacna2d3*^*p177*^*/*+ larvae are shown in blue and *cacna2d3*^*p177*^/*cacna2d3*^*sa16051*^ trans-heterozygotes are shown in red. (K) Mean acoustic startle habituation percentage. (I, K) ***p<0.001, ****p<0.0001, Mann-Whitney test versus wild-type. For all panels, the number of larvae analyzed is shown below each bar. Error bars indicate SEM.

Although WGS analysis and homozygosity mapping show the *cacna2d3* mutation to be strongly linked to the *dory* ^*p177*^ habituation defect, they do not prove a causal relationship. To address causality, we examined two additional *cacna2d3* mutant alleles obtained from the Zebrafish Mutation Project of the Sanger Center [36]. *cacna2d3*^*sa16189*^ harbors a nonsense mutation at codon 231 of 1095, upstream of both the VWA functional domain and the *δ-3* subunit (Fig 2B). *cacna2d3*^*sa16051*^ harbors a nonsense mutation at codon 697, downstream of the VWA functional domain but upstream of the *δ-3* subunit (Fig 2C).

The mean acoustic startle responsiveness of wild-type, *cacna2d3*^*sa16189*^*/+* heterozygotes, and *cacna2d3*^*sa16189*^*/+* mutants to each stimulus of the habituation assay is shown in Fig 2D. Wild-type and *cacna2d3*^*sa16189*^*/+* heterozygous siblings showed a rapid reduction in SLC startle response frequency and stereotypically habituated by 72.5% and 76.7%, respectively (Fig 2E). In contrast, *cacna2d3*^*sa16189*^ homozygous mutants exhibited weak startle habituation of 12.4% (Fig 2E; wild-type siblings n = 19, *cacna2d3*^*sa16189*^*/+* n = 39, *cacna2d3*^*sa16189*^ n = 33). The mean acoustic startle responsiveness of wild-type, *cacna2d3*^*sa16051*^*/+* heterozygotes, and *cacna2d3*^*sa16051*^*/+* mutants to each stimulus of the habituation assay is shown in Fig 2F. Wild-type and *cacna2d3*^*sa16051*^*/+* heterozygous siblings showed a rapid reduction in startle initiation and habituated by 89.8% and 86.0%, respectively, while *cacna2d3*^*sa16051*^ mutants showed weak habituation of 19.9% (Fig 2G; wild-type siblings n = 21, *cacna2d3*^*sa16051*^*/+* n = 51, *cacna2d3*^*sa16051*^ n = 20). Habituation defects linked to three independent mutant alleles of *cacna2d3* demonstrate definitively that *cacna2d3* function is required for habituation learning.

To confirm that the habituation defect in *dory*^*p177*^, now renamed *cacna2d3*^*p177*^, is indeed caused by the splice-site mutation identified in *cacna2d3*, we tested trans-heterozygous larvae for habituation. The mean acoustic startle responsiveness of *cacna2d3*^*p177*^*/+* heterozygotes and *cacna2d3*^*p177*^*/cacna2d3*^*sa16189*^ trans-heterozygotes to each stimulus of the habituation assay is shown in Fig 2H. *cacna2d3*^*p177*^*/cacna2d3*^*sa16189*^ trans-heterozygotes showed 39.5% habituation compared with the 80.5% habituation exhibited by siblings (Fig 2I; siblings n = 43, *cacna2d3*^*p177*^*/cacna2d3*^*sa16189*^ n = 53). The mean acoustic startle responsiveness of *cacna2d3*^*p177*^*/+* heterozygotes and *cacna2d3*^*p177*^*/cacna2d3*^*sa16051*^ trans-heterozygotes to each stimulus of the habituation assay is shown in Fig 2J. *cacna2d3*^*p177*^*/cacna2d3*^*sa16051*^ trans-heterozygotes showed 39.0% habituation compared to the 87.8% habituation exhibited by siblings (Fig 2K; siblings n = 50, *cacna2d3*^*p177*^*/cacna2d3*^*sa16051*^ n = 44). Together, these data indicate that the *cacna2d3*^*p177*^ habituation defect is caused by the unique T to C substitution within the splice donor sequence of intron 2–3 of *cacna2d3*.

### *cacna2d3* regulates the innate acoustic startle threshold

**Fig 3:**
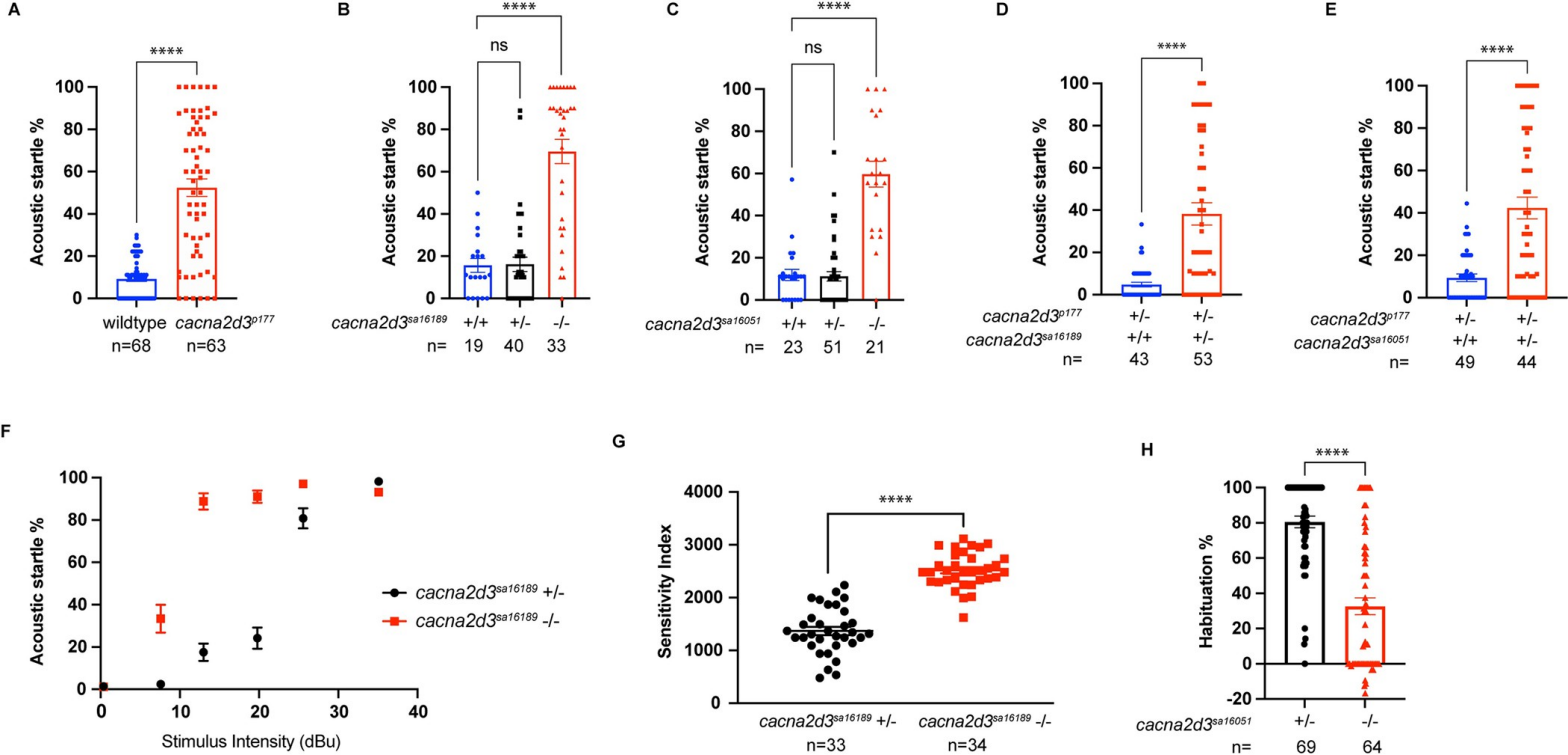
The startle threshold is reduced in *cacna2d3* mutants. (A-E) Acoustic startle (SLC) responsiveness of *cacna2d3* mutants to the 10, low-level acoustic stimuli presented during the “sensitivity” phase of the habituation assay. Startle response of (A) wild-type (shown in blue) and *cacna2d3*^*p177*^ mutants (shown in red). Startle response of (B) *cacna2d3*^*sa16189*^ wild-type (shown in blue), heterozygous (shown in black) and mutant larvae (shown in red). Startle response of (C) *cacna2d3*^*sa16051*^ wild-type (shown in blue), heterozygous (shown in black) and mutant larvae (shown in red). (B, C) ****p<0.0001, one-way ANOVA with Dunnett’s multiple comparison test. Startle response of (D) *dory*^*p177*^*/+* sibling larvae (shown in blue) and *cacna2d3*^*p177*^/ *cacna2d3*^*sa16189*^ trans-heterozygous larvae (shown in red). Startle response of *dory*^*p177*^*/+*; siblings (shown in blue) and *cacna2d3*^*p177*^/*cacna2d3*^*sa16051*^ trans-heterozygous larvae (shown in red). (A, D, E) ****p<0.0001, unpaired t-test with Welch’s correction versus wild-type. (F) Startle frequency for 30 trials at 6 stimulus intensities. *cacna2d3*^*sa16189*^*/+* heterozygotes are in black and *cacna2d3*^*sa16189*^ mutants are in red. (G) Mean startle sensitivity indices. ****p<0.0001, unpaired t-test with Welch’s correction versus heterozygotes. (H) Mean acoustic startle habituation percentage from a cross of *cacna2d3*^*sa16051*^*/+* heterozygotes and *cacna2d3*^*sa16051*^ mutants with a lowered acoustic intensity. *cacna2d3*^*sa16051*^*/+* heterozygotes are shown in black, and homozygous *cacna2d3*^*sa16051*^ mutants are shown in red. ****p<0.0001, unpaired t-test with Welch’s correction versus heterozygotes. For all panels, the number of larvae analyzed is shown below each bar. Error bars indicate SEM.

In the course of habituation analyses, we noted unusual sensitivity of the mutant larvae during the sensitivity (low intensity stimulus) phase of the assay. Compared to the low response rate observed in wild-type (TL) larvae, *cacna2d3*^*p177*^ mutants exhibited a marked increase in SLC startle responsiveness to subthreshold low-level intensity stimuli (Fig 3A; 9.3% SLC response rate wild-type larvae n = 68, compared to 52.4% in *cacna2d3*^*p177*^ homozygotes n = 63). Similarly, *cacna2d3*^*sa16189*^ mutants showed 70% SLC startle responsiveness to subthreshold low-intensity stimuli, contrasted with the 16% SLC startle responsiveness of the wild-type and heterozygous siblings (Fig 3B; wild-type siblings n = 19, *cacna2d3*^*sa16189*^*/+* n = 40, *cacna2d3*^*sa16189*^ n = 33). *cacna2d3*^*sa16051*^ mutants showed 60% SLC startle responsiveness compared to 11% of their wild-type and heterozygous siblings (Fig 3C; wild-type siblings n = 23 *cacna2d3*^*sa16051*^*/+* n = 51, *cacna2d3*^*sa16051*^ n = 21). This increased SLC startle sensitivity was also observed in *cacna2d3*^*p177*^*/cacna2d3*^*sa16189*^ trans-heterozygotes, which showed 38.2% SLC startle responsiveness compared to 4.8% of their siblings (Fig 3D; siblings n = 43, *cacna2d3*^*p177*^*/cacna2d3*^*sa16189*^ n = 53) and in *cacna2d3*^*p177*^*/cacna2d3*^*sa16051*^ trans-heterozygotes, which showed 42.4% SLC startle responsiveness compared to 9.4% of siblings (Fig 3E; siblings n = 49, *cacna2d3*^*p177*^*/cacna2d3*^*sa16051*^ n = 44). SLC startle sensitivity defects in trans-heterozygotes were consistently less pronounced than in homozygous larvae, possibly due to differences in genetic backgrounds between the strains in which these alleles were maintained. Collectively, these results are consistent with the notion that loss of *cacna2d3* leads to a lower threshold of the startle response.

To test this hypothesis further, we subjected *cacna2d3*^*sa16189*^ mutants and their heterozygous siblings to varying intensities of acoustic stimuli at non-habituating intervals and measured SLC startle responsiveness. This analysis revealed that intensities as low as 7.6 dB cause a significant increase in SLC startle responsiveness of 33.4% in *cacna2d3*^*sa16189*^ mutants, compared to 2.4% in heterozygous siblings (Fig 3F; *cacna2d3*^*sa16189*^*/+* n = 33, *cacna2d3*^*sa16189*^ n = 33). At 13 dB, the SLC startle responsiveness in *cacna2d3*^*sa16189*^ mutants reaches 88.8%, a level that their heterozygous siblings do not reach until 25.6 dB. To quantify the severity of this hypersensitivity phenotype, we calculated the startle sensitivity index by plotting the startle frequency of each larva across the 30-stimulus assay and measuring the area under the resulting curves. *cacna2d3*^*sa16189*^ homozygous mutants exhibited significant hypersensitivity compared to their heterozygous siblings (Fig 3G; *cacna2d3*^*sa16189*^*/+* n = 33, *cacna2d3*^*sa16189*^ n = 34).

In wildtype larvae, habituation to acoustic stimuli is inversely proportional to stimulus intensity; in other words, habituation is more robust in response to weaker stimuli [3]. We hypothesized that reducing stimulus intensity would restore the ability of *cacna2d3* mutants to habituate. To test this hypothesis, we modified the habituation assay to use acoustic stimuli empirically determined to elicit SLCs ~25% of the time in wildtype larvae. When subjected to these intermediate reduced-intensity stimuli, cacna*2d3*^*sa16051*^ mutants still failed to habituate fully, with *cacna2d3*^*sa16051*^ mutants exhibiting 32.6% habituation compared to 80.5% in heterozygous siblings (Fig 3H; *cacna2d3*^*sa16051*^*/+* n = 69, *cacna2d3*^*sa16051*^ n = 64). Collectively, these data show that, in addition to its role in habituation, *cacna2d3* is required for establishing or maintaining acoustic startle thresholds.

### *cacna2d3* controls latency of the acoustic startle response

**Fig 4:**
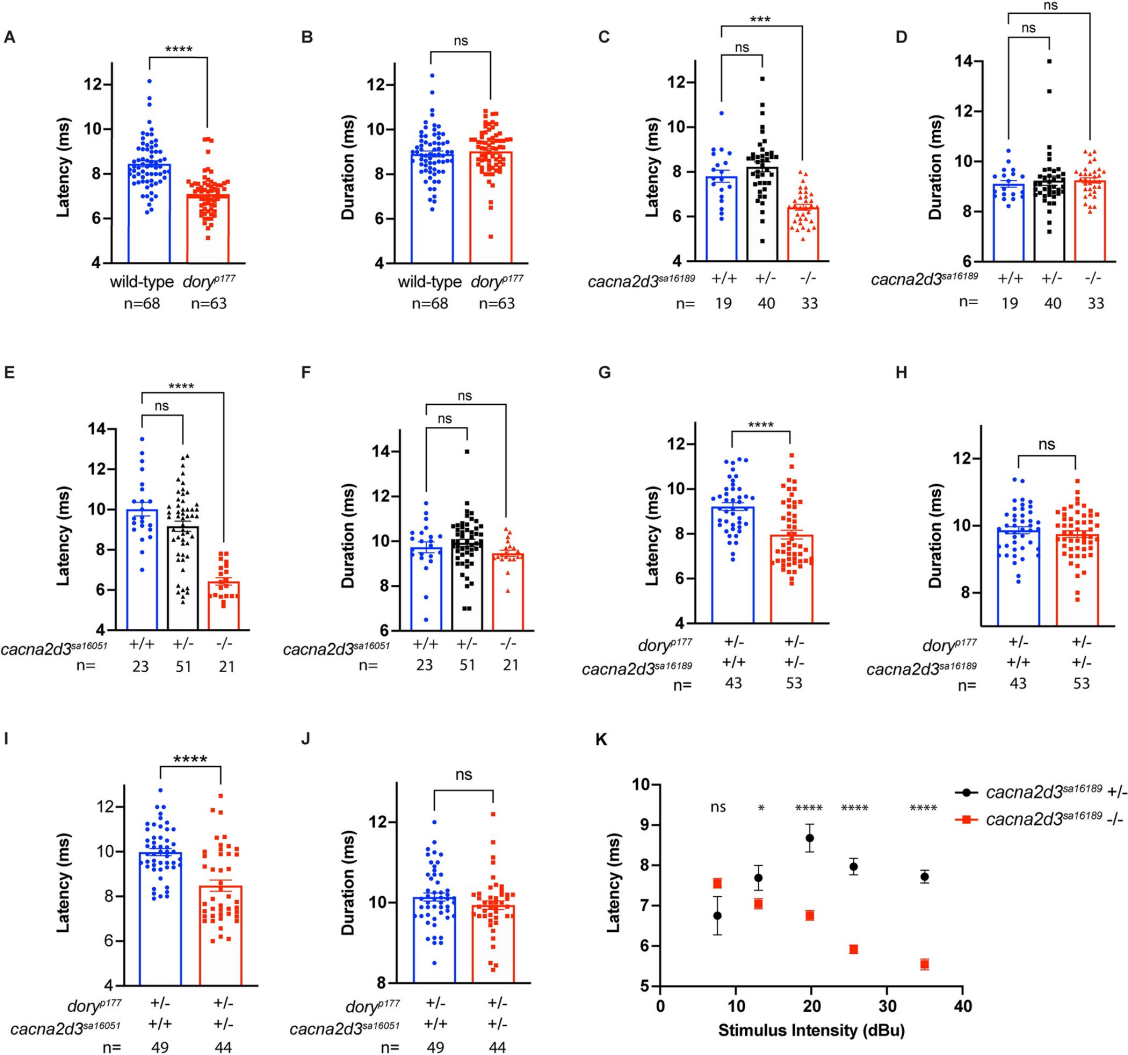
*cacna2d3* mutants and trans-heterozygotes exhibit decreased startle latency. Startle kinematic analysis of C-turn latency and C-turn duration. (A) Latency and (B) duration analysis and of wild-type (shown in blue) and *cacna2d3*^*p177*^ mutants (shown in red). (A-B) ns, ****p<0.0001, Mann-Whitney test versus wild-type. (C) Latency and (D) duration analysis of *cacna2d3*^*sa16189*^ wild-type (shown in blue), heterozygous (shown in black) and mutant larvae (shown in red). (E) Latency and (F) duration analysis of *cacna2d3*^*sa16051*^ wild-type (shown in blue), heterozygous (shown in black) and mutant larvae (shown in red). (C, E) ***p<0.001, ****p<0.0001, one-way ANOVA with Dunnett’s multiple comparison test versus wild-type.

To investigate the motor function of mutant larvae, we assessed their SLC kinematics. The SLC maneuver executed in response to high intensity acoustic stimuli is defined by kinematic parameters within a well-defined range of values. These parameters include C-turn initiation latency and C-turn duration [21]. *cacna2d3*^*p177*^ mutants showed a marked reduction in C-turn latency compared to wild-type (TL) larvae (Fig 4A) but no difference in C-turn duration (Fig 4B). SLC kinematic analysis of the *cacna2d3*^*sa16189*^ and *cacna2d3*^*sa16051*^ mutants revealed that both mutants also showed a significant reduction in C-turn latency (Fig 4C and 4E, respectively), but no change in C-turn duration (Fig 4D and 4F, respectively). Lastly, in order to confirm a causative role for *cacna2d3* in regulating C-turn latency, but not C-turn duration, we evaluated the startle kinematics of the trans-heterozygotes in the complementation analysis, which revealed that *cacna2d3*^*p177*^*/ cacna2d3*^*sa16189*^ *larvae* exhibited reduced C-turn latency (Fig 4G) and no change in C-turn duration (Fig 4H). Similarly, *cacna2d3*^*p177*^*/cacna2d3*^*sa16051*^ trans-heterozygotes showed reduced C-turn latency (Fig 4I) with no change in C-turn duration (Fig 4J). The decreased latency of the SLC is likely due to decreased startle threshold [37], which leads them to initiate the escape maneuver more rapidly than their wild-type and heterozygous siblings. Despite these differences, these kinematic parameters are still within the range previously used to define SLC responses [21] and are consistent with a normal motor function controlling escape responses in *cacna2d3* mutants.

To investigate the relationship between C-turn latency and startle sensitivity, we analyzed the SLC turn latency of *cacna2d3*^*sa16189*^ mutants and their heterozygous siblings in response to varying intensities of acoustic stimuli, beginning at 7.6 dB. At 7.6 dB, SLC turn latency was indistinguishable between *cacna2d3*^*sa16189*^ mutants and heterozygotes (Fig 4K); however, startle responsiveness was still significantly higher in *cacna2d3*^*sa16189*^ mutants at this stimulus intensity (Fig 3F). This finding unlinks hypersensitivity in *cacna2d3*^*sa16189*^ mutants from the latency to execute the escape maneuver.

(D, F) ns, Kruskall-Wallis test with Dunn’s multiple comparisons test versus wild-type. (G) Latency and (H) duration analysis of *cacna2d3*^*p177*^*/+* sibling larvae (shown in blue) and *cacna2d3*^*p177*^/*cacna2d3*^*sa16189*^ trans-heterozygous larvae (shown in red). (I) Latency and (J) duration analysis of *cacna2d3*^*p177*^*/+* siblings (shown in blue) and *cacna2d3*^*p177*^/*cacna2d3*^*sa16051*^ trans-heterozygous larvae (shown in red). (G-I) ns, ****p<0.0001, unpaired t-test with Welch’s correction versus *cacna2d3*^*p177*^/+ siblings. (J) ns, Mann-Whitney test versus *cacna2d3*^*p177*^*/+* siblings. (K) SLC turn latency versus stimulus intensity for *cacna2d3*^*sa16189*^*/+* heterozygotes (in black) and *cacna2d3*^*sa16189*^ mutants (in red); 30 trials. Mean was compared using unpaired t-test with Welch’s correction versus *cacna2d3*^*sa16189*^/+ siblings. Sample sizes are as follows: 0.3 dB, *cacna2d3*^*sa16189*^/+ n = 2, *cacna2d3*^*sa16189*^ n = 2 (not reported on graph); 7.6 dB: *cacna2d3*^*p177*^/+ n = 4, *cacna2d3* ^*sa16189*^ n = 54; 13 dB: *cacna2d3* ^*sa16189*^/+ n = 29, *cacna2d3* ^*sa16189*^ n = 148; 19.8 dB: *cacna2d3*^*sa16189*^/+ n = 40, *cacna2d3*^*sa161897*^ n = 149; 25.6 dB: *cacna2d3*^*sa16189*^/+ n = 129, *cacna2d3*^*sa16189*^ n = 162; and 35 dB: *cacna2d3*^*sa16189*^/+ n = 161, *cacna2d3*^*sa16189*^ n = 153. ns, *p<0.05, ****p<0.0001, Mann-Whitney test versus *cacna2d3*^*sa16189*^. For panels A-J, the number of larvae analyzed is shown below each bar. For all panels, error bars indicate SEM.

## Discussion

Habituation is a fundamental form of learning that is conserved across species [2,3,38]. It is defined as a learning process in which an organism’s responsiveness to a given stimulus progressively declines with repeated exposure. In humans, aberrant habituation is a hallmark of many behavioral disorders that exhibit cognitive dysfunction, including ASDs [11–13], schizophrenia [15], and ADHD [17]. The etiologies of these disorders are under intense scrutiny, and there is a critical need to identify genes important for habituation as candidate therapeutic targets for these disorders. Whole genome sequence analysis of the *dory/cacna2d3*^*p177*^ mutant line, obtained in a forward-genetic screen for habituation mutants [27], led us to uncover a previously unknown role for *cacna2d3* in habituation to acoustic stimuli in vertebrates, and in establishing or maintaining a baseline innate startle threshold, thus altering sensitivity to acoustic stimuli.

*cacna2d3* encodes the calcium voltage-gated channel auxiliary subunit α2δ3. α2δ subunits function at the presynaptic terminal by strengthening the coupling between calcium influx and neurotransmitter release [34]. The α2 and δ3 subunits are generated through proteolytic cleavage of a precursor protein encoded by *cacna2d3* [32]. In the ER, a GPI anchor is added to the δ3 protein, which anchors the subunit to the cell membrane [39]. Cacna2d3 is highly conserved across vertebrates, including zebrafish (S1 Fig), particularly in the VWA domain that is involved in protein-protein interactions via a metal ion adhesion (MIDAS) motif [32] and is important for trafficking of VGCCs [40]. α2δ subunit function is not limited to the pre-synapse. α2δ subunits, in association with VGCCs, contribute to dendritic computations [41], shaping the action potential in the axon [42], and mediating calcium-channel dependent gene regulation [43]. In the context of the Mauthner neuron, dendritic calcium signaling determines startle probability [9].

In addition to impaired habituation, we have documented enhanced sensitivity to acoustic stimuli in *cacna2d3* mutants (Fig 3A–3E). This is evidenced by an increased sensitivity index, suggesting their innate startle threshold is reduced (Fig 3F and 3G). The innate threshold for the startle response is an important mechanism for regulating threat evasion [37]; reduction of this threshold and the subsequent hypersensitivity to acoustic stimuli has been strongly linked to ASD [13] and anxiety in humans [44]. Deficits in acoustic sensitivity and habituation frequently co-segregate, possibly due to partially overlapping circuitry and/or genetic pathways that regulate these processes. Notably, our previous screen identified mutants in which habituation deficits occurred independently of acoustic hypersensitivity [27] and mutants in which acoustic hypersensitivity occurred independently of habituation deficits [37], consistent with the notion that these behavior are controlled by partially independent molecular pathways. Future studies will ask how the hypersensitivity observed in *cacna2d3* mutants contributes to their habituation defect, thus contributing to our understanding of the relationship between acoustic sensitivity and habituation.

We also observed decreased SLC latency in all three mutant alleles (Fig 4A, 4C and 4E), suggesting a link between decreased SLC latency and hypersensitivity. We explored this relationship by comparing the SLC latency and startle responsiveness of *cacna2d3*^*sa16189*^ mutants and heterozygous siblings at several stimulus intensities. This analysis unlinked SLC latency from startle responsiveness (Figs 3 and 4). Previous work has shown that SLCs are all-or-nothing responses, and that increasing stimulus intensity increases the probability of eliciting an SLC response but does not alter the kinematics of the response [21]. In heterozygous larvae, we show that the startle latency is not altered proportionally with stimulus intensity. While we do find that hypersensitive mutants have a decreased latency, latency is not modulated with respect to stimulus intensity and should not be used as a proxy for how a stimulus is perceived.

It is worth noting that several behavioral parameters measured in our assays were less severely affected in trans-heterozygous combinations that included the *cacna2d3*^*p177*^ allele relative to homozygotes. This intriguing observation was unexpected since all three alleles are predicted to encode functional nulls that lack the δ subunit, thought to be required for the function of α2δ3 [39,45]. Since the *cacna2d3*^*p177*^ allele was generated in a different laboratory strain than the other two alleles, the observed normalization of phenotypes in trans-heterozygotes may be due to an overall increase in heterozygosity. Inter-strain genomic variability is well documented in zebrafish [46], as is strain-specific variability in behavioral phenotypes [47]. An inter-strain hybrid is expected to differ from each of the parent strains due to heterozygosity at many loci, some of which may contribute to regulation of behavior and perhaps interact with *cacna2d3* to modulate its function.

Mutations in the *C*. *elegans* ortholog of *cacna2d3*, *unc-36*, have been linked with impaired habituation to tactile stimuli, as well as increased tactile sensitivity [48]. In combination with our findings that *cacna2d3* mutant zebrafish show impaired habituation to acoustic stimuli (Figs 1E, 2B and 2F), these data suggest a strong functional conservation of *cacna2d3* in habituation learning. In contrast to *cacna2d3* mutant zebrafish larvae, adult mice with *CACNA2D3* dysfunction exhibit reduced ASR when presented with acoustic stimuli and have a higher startle threshold [35]. This difference may reflect different functions of Cacna2d3 in adults vs. larvae. Alternatively, it may be due to the fact that zebrafish detect acoustic stimuli both via the hair cells in the otic vesicles, like mammals, and through the lateral line hair cells, which detect vibrations in the water and encode them as mechanosensory stimuli [49].

Remarkably, *CACNA2D3* mutant mice show a marked increase in tactile startle responsiveness when presented with stimuli elicited by air puffs. The increased startle responsiveness to tactile stimuli in mice and *C*. *elegans* is of particular interest, as tactile hypersensitivity has been linked to anxiety, autism, and migraines [10,50]. It may be that, similar to the tactile hypersensitivity observed in *C*. *elegans* [48] and mice [35], hypersensitivity observed in *cacna2d3* mutant zebrafish is due to a combination of acoustic- and mechanosensory-driven aberrant startle responses. Accessibility of the lateral line hair cells to pharmacological manipulation and ablation offers an effective strategy to test this hypothesis in the future [51].

## Conclusions

Our findings identify essential functions for zebrafish *cacna2d3* in acoustic startle sensitivity and habituation. The high degree of sequence conservation suggests strong conservation of Cacna2d3 protein functions from zebrafish to human and supports the value of *cacna2d3* mutant zebrafish as a clinically relevant model for elucidating the underlying mechanisms of sensory filtering impairments associated with prevalent neurodevelopmental disorders.

## Methods

### Generation and maintenance of zebrafish

Zebrafish (Danio rerio) were maintained according to established methods [52]. All experimental protocols using zebrafish were approved by the University of Wisconsin Animal Care and Use Committee and carried out in accordance with the institutional animal care protocols. Embryos were generated from natural matings of adult Tüpel long fin (TL), and adults carrying the *dory/cacna2d3*^*p177*^, *cacna2d3*^*sa16051*^, and *cacna2d3*^*sa16189*^ alleles, respectively. TL wild-type animals were used for behavioral comparisons because the *dory*^*p177*^ allele was maintained by backcrossing to the TL background. Embryos were raised in E3 media at 28°C on a 14 hr/10 hr light/dark cycle through 5 dpf as previously described [53,54]. 5 dpf larvae were analyzed for behavior in E3.

### Genotyping

To genotype larvae, we developed dCAPS assays using the dCAPS program (http://helix.wustl.edu/dcaps/dcaps.html) to design appropriate primers [55] for the *dory*^*p177*^ and *cacna2d3*^*sa16051*^ alleles. Primers for the *cacna2d3*^*sa16189*^ allele were designed using Primer3 [56]. Primer sequences, PCR conditions, and restriction endonucleases used for digestion are outlined in **Table 1**. All genotyping was performed after behavioral experiments. *cacna2d3*^*sa16189*^ larvae used in sensitivity assay were genotyped using the KASP method with proprietary primer sequences (LGC Genomics).

**Table 1:** Genotyping primers and genotyping conditions.

Allele	Primers	Annealing temperature	Restriction endonuclease	Product type	Product digestion	DNA fragments after digestion
*dory* ^*p177*^	Fwd: 5′ TCC CAC ACG GTT TAG TCA TAC A 3′	54°C	RsaI	PCR product containing the mutation	unaffected	220bp
	dCAPS Rev: 5′ AGA GAG AAG GGG AA**G** T 3′			PCR product derived from WT template	cleaved	204bp +16bp
*cacna2d3* ^*sa16051*^	Fwd: 5′ GTT TGG CCA CAA TGT CCT TT 3′	57°C	RsaI	PCR product containing the mutation	cleaved	147bp + 24bp
	dCAPS Rev: 5′ GTG ACC ACA GCA TCA AAC AGA ACG T 3′			PCR product derived from WT template	unaffected	171bp
*cacna2d3* ^*sa16189*^	Fwd: 5′ GCA TCT GCA AGC TTA ATG ATT TT 3′	52°C	EcoRV	PCR product containing the mutation	cleaved	129bp + 40bp
	Rev: 5′ TTT CTG CAA TCA AAT GCA ATG 3′			PCR product derived from WT template	unaffected	169bp

Genotyping primers, annealing temperatures, restriction endonuclease used for digestion, and expected band fragment sizes are listed. A mismatch (marked in bold) has been introduced into the reverse primer for the *dory*^*p177*^ allele that creates an RsaI restriction enzyme site in the amplified product from the wild-type DNA template. Similarly, a mismatch (marked in bold) has been introduced into the reverse primer for the *cacna2d3*^*sa16051*^ allele that creates an RsaI restriction enzyme site in the amplified product from the mutant DNA template.

### Behavioral analyses

On the day of habituation behavioral testing and acoustic sensitivity analysis, larvae were held in 60mm-wide Petri dishes with 25 larvae in 10mL E3, kept on a white light box for at least 30 minutes, and then transferred to a 6x6 grid. Startle behavior was elicited using an automated behavioral platform in which the intensity and timing of acoustic stimuli could be controlled [21,28]. Startle responses were elicited with a minishaker (Brüel & Kjær, Model 4810). For the habituation assays, the acoustic stimuli were of 3 millisecond duration, with 1000 Hz waveforms, at either low-level, subthreshold intensity identified empirically to elicit SLCs ~10% of the time in wild-type larvae or above threshold, high intensity identified empirically to elicit SLCs ~80% of the time in wild-type larvae. For the experiment examining the relationship between the habituation defect in *cacna2d3* mutants and their behavioral hypersensitivity, we used an intermediate level acoustic stimulus selected empirically to elicit SLCs ~25% of the time in wild-type larvae. This intensity was selected to allow for the analysis of startle responsiveness in heterozygotes and mutants, and to determine whether mutant larvae habituation would be improved at a lower acoustic stimulus intensity.

The habituation assay consists of multiple phases, each designed to assess different parameters of short-term acoustic startle habituation [28]. To evaluate acoustic sensitivity, low-level “subthreshold” intensity stimuli were presented at a 30 second interstimulus interval (ISI) (stimuli 1–10), eliciting ~10% startle responses in wild-type larvae. This phase is followed by a 30 second break before moving on to the next phase. To evaluate short-term startle habituation, high-intensity stimuli were presented at 30 second ISI during the “pre-habituation” phase (stimuli 11–20) and at 3 second ISI during the “habituation” phase (stimuli 21–50). There is a 30 second break between the “pre-habituation” phase and the “habituation” phase of the assay. The degree to which larvae habituate was calculated by comparing the average frequency of startle responsiveness of an individual during the pre-habituation and the last 10 stimuli of the habituation phases (stimuli 41–50) [28].

Startle responses were captured at 1000 frames per second with a MotionPro Y4 video camera (Integrated Design Tools) with a 50 mm macro lens (Sigma Corporation of America) at 512 x 512 pixel resolution. We used FLOTE to analyze startle responses in an experimenter-independent, automated manner [21]. FLOTE tracks the position of individual larvae frame by frame and characterizes locomotor maneuvers (e.g. C-bend, routine turn, swim, etc) according to predefined kinematic parameters that distinguish these maneuvers. We used a custom R-script to run analysis on behavior data generated by FLOTE to calculate habituation and analyze kinematic data, which allowed for an additional level of experimenter-independent, automated analysis. For startle behavior, we report data representing the short-latency C-bend (SLC) startle response. When testing individual larvae for habituation, animals that exhibited a startle response of <40% to acoustic stimuli during the “pre-habituation” phase were classed as non-responders and excluded from analysis. For kinematic data, we report the SLC response of larvae during the 10 high-intensity stimuli given during the “pre-habituation” phase of the habituation assay.

For the generation of sensitivity index calculations, *cacna2d3*^*sa16189*^ homozygous mutants were crossed with *cacna2d3*^*sa16189*^*/+* heterozygous carriers. Larvae were tested for acoustic behavioral sensitivity at 5dpf and analyzed using FLOTE software as described previously [21,37]. Briefly, larvae were presented with a total of 30 acoustic stimuli: 5 trials of 6 different stimulus intensities at the following decibel levels: 0.3 dB, 7.6 dB, 13 dB, 19.8 dB, 25.6 dB, and 35 dB. Each stimulus was separated with a 40-second ISI. Percent startle for each larva was recorded at each stimulus intensity. Sensitivity index was calculated for each larva by calculating the area under the curve of percent startle vs. stimulus intensity using Prism (GraphPad). Stimulus intensities were calibrated using a PCB Piezotronics accelerometer (#355B04) and signal conditioner (#482A21). Voltage outputs were converted to dBu using the formula dBu = 20* log (V/0.775).

For the analysis of the C-turn latency in response to lowered acoustic intensity stimuli, we report the SLC turn latency of larvae at the following decibel levels: 0.3 dB, 7.6 dB, 13 dB, 19.8 dB, 25.6 dB, and 35 dB. For each stimulus intensity, the mean SLC turn latency was calculated and compared between genotypes using a Mann-Whitney test versus *cacna2d3*^*sa16189*^/+ siblings.

### Whole genome sequencing and analysis

Positional cloning was performed as previously described [27]. A pool of 64 behaviorally identified *dory* mutant larvae was collected, genomic DNA (gDNA) was extracted, and gDNA libraries were prepared. gDNA was sequenced with 100-bp paired-end reads on the Illumina HiSeq 2000 platform, and homozygosity analysis was done using 463,379 SNP markers identified by sequencing gDNA from ENU- mutagenized TL and WIK males as described previously [27]. SnapGene software (from Insightful Science; available at snapgene.com) was used for DNA and protein sequence analysis.

### Statistics

All graph generation and statistical analyses, including calculation of means and SEM, were performed using Graphpad Prism (www.graphpad.com). D’Agostino and Pearson normality test was used to test whether data were normally distributed. If data were normally distributed, significance was assessed using t-tests with Welch’s correction or ANOVA with Dunnet’s multiple comparisons test. If data were not normally distributed, Mann-Whitney test or Kruskall-Wallis test with Dunn’s multiple comparisons test was used.

## Supporting information

S1 Fig Protein conservation between humans and zebrafish. The alignment between human CACNA2D3 and zebrafish Cacna2d3 protein sequence was generated using the local alignment algorithm (Smith-Waterman) in SnapGene software. Human CANCA2D3 and zebrafish Cacna2d3 proteins share 76.5% amino acid identity and 87.6% similarity along the length of the protein. In the Von Willebrand Factor A (VWA) functional domain, the proteins share 89.4% amino acid identity and 93.4% similarity. Human CACNA2D3 is designated by row 1 and zebrafish Cacna2d3 is designated by row 2. VWA domain designated by blue box. | = identical amino acid;: = similar amino acid;. = not similar amino acid.
(TIF)

S2 Fig Predicted outcomes of *dory*^*p177*^, a thymine to cytosine single base pair substitution at the splice donor site. Predicted amino acid sequences encoded by *dory*^*p177*^ if the mutation causes retention of intron 2–3 (A) and if it causes skipping of exon 2 (B).
(TIF)

S1 Table (XLSX)

## References

[1] Ramaswami M. Network plasticity in adaptive filtering and behavioral habituation. Neuron. 2014;82(6):1216–29. Epub 2014/06/20. doi: 10.1016/j.neuron.2014.04.035 . 24945768

[2] Groves PM, Thompson RF. Habituation: a dual-process theory. Psychol Rev. 1970;77(5):419–50. . 4319167 10.1037/h0029810

[3] Rankin CH, Abrams T, Barry RJ, Bhatnagar S, Clayton DF, Colombo J, et al. Habituation revisited: an updated and revised description of the behavioral characteristics of habituation. Neurobiol Learn Mem. 2009;92(2):135–8. Epub 2008/10/16. doi: 10.1016/j.nlm.2008.09.012 ; PubMed Central PMCID: PMC2754195. 18854219 PMC2754195

[4] Ardiel EL, Yu AJ, Giles AC, Rankin CH. Habituation as an adaptive shift in response strategy mediated by neuropeptides. NPJ Sci Learn. 2017;2:9. Epub 2017/08/18. doi: 10.1038/s41539-017-0011-8 ; PubMed Central PMCID: PMC6161508. 30631455 PMC6161508

[5] Simons-Weidenmaier NS, Weber M, Plappert CF, Pilz PK, Schmid S. Synaptic depression and short-term habituation are located in the sensory part of the mammalian startle pathway. BMC Neurosci. 2006;7:38. Epub 2006/05/11. doi: 10.1186/1471-2202-7-38 ; PubMed Central PMCID: PMC1479352. 16684348 PMC1479352

[6] Aljure E, Day JW, Bennett MV. Postsynaptic depression of Mauthner cell-mediated startle reflex, a possible contributor to habituation. Brain Res. 1980;188(1):261–8. Epub 1980/04/21. doi: 10.1016/0006-8993(80)90574-0 7370756

[7] Castellucci VF, Kandel ER. A quantal analysis of the synaptic depression underlying habituation of the gill-withdrawal reflex in Aplysia. Proc Natl Acad Sci U S A. 1974;71(12):5004–8. Epub 1974/12/01. doi: 10.1073/pnas.71.12.5004 ; PubMed Central PMCID: PMC434028. 4373738 PMC434028

[8] Weber M, Schnitzler HU, Schmid S. Synaptic plasticity in the acoustic startle pathway: the neuronal basis for short-term habituation? Eur J Neurosci. 2002;16(7):1325–32. Epub 2002/10/31. doi: 10.1046/j.1460-9568.2002.02194.x . 12405993

[9] Marsden KC, Granato M. In Vivo Ca(2+) Imaging Reveals that Decreased Dendritic Excitability Drives Startle Habituation. Cell Rep. 2015;13(9):1733–40. Epub 2015/12/15. doi: 10.1016/j.celrep.2015.10.060 ; PubMed Central PMCID: PMC4680997. 26655893 PMC4680997

[10] McDiarmid TA, Bernardos AC, Rankin CH. Habituation is altered in neuropsychiatric disorders-A comprehensive review with recommendations for experimental design and analysis. Neurosci Biobehav Rev. 2017;80:286–305. Epub 2017/06/06. doi: 10.1016/j.neubiorev.2017.05.028 . 28579490

[11] Perry W, Minassian A, Lopez B, Maron L, Lincoln A. Sensorimotor gating deficits in adults with autism. Biol Psychiatry. 2007;61(4):482–6. Epub 2006/02/08. doi: 10.1016/j.biopsych.2005.09.025 . 16460695

[12] Ornitz EM, Lane SJ, Sugiyama T, de Traversay J. Startle modulation studies in autism. J Autism Dev Disord. 1993;23(4):619–37. Epub 1993/12/01. doi: 10.1007/BF01046105 . 8106303

[13] Takahashi H, Komatsu S, Nakahachi T, Ogino K, Kamio Y. Relationship of the Acoustic Startle Response and Its Modulation to Emotional and Behavioral Problems in Typical Development Children and Those with Autism Spectrum Disorders. J Autism Dev Disord. 2016;46(2):534–43. Epub 2015/09/13. doi: 10.1007/s10803-015-2593-4 . 26362152

[14] Castren M, Paakkonen A, Tarkka IM, Ryynanen M, Partanen J. Augmentation of auditory N1 in children with fragile X syndrome. Brain Topogr. 2003;15(3):165–71. Epub 2003/04/23. doi: 10.1023/a:1022606200636 . 12705812

[15] Braff DL, Geyer MA. Sensorimotor gating and schizophrenia. Human and animal model studies. Arch Gen Psychiatry. 1990;47(2):181–8. Epub 1990/02/01. doi: 10.1001/archpsyc.1990.01810140081011 . 2405807

[16] Agostino R, Berardelli A, Cruccu G, Pauletti G, Stocchi F, Manfredi M. Correlation between facial involuntary movements and abnormalities of blink and corneal reflexes in Huntington’s chorea. Mov Disord. 1988;3(4):281–9. Epub 1988/01/01. doi: 10.1002/mds.870030401 . 2974927

[17] Jansiewicz EM, Newschaffer CJ, Denckla MB, Mostofsky SH. Impaired habituation in children with attention deficit hyperactivity disorder. Cogn Behav Neurol. 2004;17(1):1–8. Epub 2004/06/24. doi: 10.1097/00146965-200403000-00001 . 15209220

[18] Penders CA, Delwaide PJ. Blink reflex studies in patients with Parkinsonism before and during therapy. J Neurol Neurosurg Psychiatry. 1971;34(6):674–8. Epub 1971/12/01. doi: 10.1136/jnnp.34.6.674 ; PubMed Central PMCID: PMC1083500. 5158781 PMC1083500

[19] Bock RD, Goldberger L. Tonic, phasic and cortical arousal in Gilles de la Tourette’s syndrome. J Neurol Neurosurg Psychiatry. 1985;48(6):535–44. Epub 1985/06/01. doi: 10.1136/jnnp.48.6.535 ; PubMed Central PMCID: PMC1028369. 3859582 PMC1028369

[20] Coppola G, Di Lorenzo C, Schoenen J, Pierelli F. Habituation and sensitization in primary headaches. J Headache Pain. 2013;14:65. Epub 2013/08/01. doi: 10.1186/1129-2377-14-65 ; PubMed Central PMCID: PMC3733593. 23899115 PMC3733593

[21] Burgess HA, Granato M. Sensorimotor gating in larval zebrafish. J Neurosci. 2007;27(18):4984–94. doi: 10.1523/JNEUROSCI.0615-07.2007 . 17475807 PMC6672105

[22] Wolman M, Granato M. Behavioral genetics in larval zebrafish: learning from the young. Dev Neurobiol. 2012;72(3):366–72. doi: 10.1002/dneu.20872 . 22328273 PMC6430578

[23] Korn H, Faber DS. The Mauthner cell half a century later: a neurobiological model for decision-making? Neuron. 2005;47(1):13–28. doi: 10.1016/j.neuron.2005.05.019 . 15996545

[24] Lingenhohl K, Friauf E. Giant neurons in the rat reticular formation: a sensorimotor interface in the elementary acoustic startle circuit? J Neurosci. 1994;14(3 Pt 1):1176–94. Epub 1994/03/01. ; PubMed Central PMCID: PMC6577542. 8120618 10.1523/JNEUROSCI.14-03-01176.1994 PMC6577542

[25] Gahtan E, Sankrithi N, Campos JB, O’Malley DM. Evidence for a widespread brain stem escape network in larval zebrafish. J Neurophysiol. 2002;87(1):608–14. Epub 2002/01/11. doi: 10.1152/jn.00596.2001 . 11784774

[26] Ogino K, Yamada K, Nishioka T, Oda Y, Kaibuchi K, Hirata H. Phosphorylation of Gephyrin in Zebrafish Mauthner Cells Governs Glycine Receptor Clustering and Behavioral Desensitization to Sound. J Neurosci. 2019;39(45):8988–97. Epub 2019/09/29. doi: 10.1523/JNEUROSCI.1315-19.2019 ; PubMed Central PMCID: PMC6832674. 31558619 PMC6832674

[27] Wolman MA, Jain RA, Marsden KC, Bell H, Skinner J, Hayer KE, et al. A genome-wide screen identifies PAPP-AA-mediated IGFR signaling as a novel regulator of habituation learning. Neuron. 2015;85(6):1200–11. Epub 2015/03/11. doi: 10.1016/j.neuron.2015.02.025 ; PubMed Central PMCID: PMC4368495. 25754827 PMC4368495

[28] Wolman MA, Jain RA, Liss L, Granato M. Chemical modulation of memory formation in larval zebrafish. Proc Natl Acad Sci U S A. 2011;108(37):15468–73. Epub 2011/08/31. doi: 10.1073/pnas.1107156108 ; PubMed Central PMCID: PMC3174630. 21876167 PMC3174630

[29] Nelson JC, Witze E, Ma Z, Ciocco F, Frerotte A, Randlett O, et al. Acute Regulation of Habituation Learning via Posttranslational Palmitoylation. Curr Biol. 2020;30(14):2729–38 e4. Epub 2020/06/06. doi: 10.1016/j.cub.2020.05.016 ; PubMed Central PMCID: PMC8446937. 32502414 PMC8446937

[30] Jain RA, Wolman MA, Marsden KC, Nelson JC, Shoenhard H, Echeverry FA, et al. A Forward Genetic Screen in Zebrafish Identifies the G-Protein-Coupled Receptor CaSR as a Modulator of Sensorimotor Decision Making. Curr Biol. 2018;28(9):1357–69 e5. Epub 2018/04/24. doi: 10.1016/j.cub.2018.03.025 ; PubMed Central PMCID: PMC5940496. 29681477 PMC5940496

[31] Bauer CS, Tran-Van-Minh A, Kadurin I, Dolphin AC. A new look at calcium channel alpha2delta subunits. Curr Opin Neurobiol. 2010;20(5):563–71. doi: 10.1016/j.conb.2010.05.007 . 20579869

[32] Dolphin AC. The alpha2delta subunits of voltage-gated calcium channels. Biochim Biophys Acta. 2013;1828(7):1541–9. Epub 2012/12/01. doi: 10.1016/j.bbamem.2012.11.019 . 23196350

[33] Dolphin AC. Voltage-gated calcium channels and their auxiliary subunits: physiology and pathophysiology and pharmacology. J Physiol. 2016;594(19):5369–90. Epub 2016/06/09. doi: 10.1113/JP272262 ; PubMed Central PMCID: PMC5043047. 27273705 PMC5043047

[34] Hoppa MB, Lana B, Margas W, Dolphin AC, Ryan TA. alpha2delta expression sets presynaptic calcium channel abundance and release probability. Nature. 2012;486(7401):122–5. doi: 10.1038/nature11033 ; PubMed Central PMCID: PMC3376018. 22678293 PMC3376018

[35] Pirone A, Kurt S, Zuccotti A, Ruttiger L, Pilz P, Brown DH, et al. alpha2delta3 is essential for normal structure and function of auditory nerve synapses and is a novel candidate for auditory processing disorders. J Neurosci. 2014;34(2):434–45. doi: 10.1523/JNEUROSCI.3085-13.2014 . 24403143 PMC6608152

[36] Sanger Institute Zebrafish Mutation Project mutant data submission [Internet]. ZFIN Direct Data Submission. 2013.

[37] Marsden KC, Jain RA, Wolman MA, Echeverry FA, Nelson JC, Hayer KE, et al. A Cyfip2-Dependent Excitatory Interneuron Pathway Establishes the Innate Startle Threshold. Cell Rep. 2018;23(3):878–87. Epub 2018/04/19. doi: 10.1016/j.celrep.2018.03.095 ; PubMed Central PMCID: PMC6642828. 29669291 PMC6642828

[38] Thompson RF, Spencer WA. Habituation: a model phenomenon for the study of neuronal substrates of behavior. Psychol Rev. 1966;73(1):16–43. Epub 1966/01/01. doi: 10.1037/h0022681 . 5324565

[39] Davies A, Kadurin I, Alvarez-Laviada A, Douglas L, Nieto-Rostro M, Bauer CS, et al. The alpha2delta subunits of voltage-gated calcium channels form GPI-anchored proteins, a posttranslational modification essential for function. Proc Natl Acad Sci U S A. 2010;107(4):1654–9. Epub 2010/01/19. doi: 10.1073/pnas.0908735107 ; PubMed Central PMCID: PMC2824380. 20080692 PMC2824380

[40] Canti C, Nieto-Rostro M, Foucault I, Heblich F, Wratten J, Richards MW, et al. The metal-ion-dependent adhesion site in the Von Willebrand factor-A domain of alpha2delta subunits is key to trafficking voltage-gated Ca2+ channels. Proc Natl Acad Sci U S A. 2005;102(32):11230–5. Epub 2005/08/03. doi: 10.1073/pnas.0504183102 ; PubMed Central PMCID: PMC1183569. 16061813 PMC1183569

[41] Kurshan PT, Oztan A, Schwarz TL. Presynaptic alpha2delta-3 is required for synaptic morphogenesis independent of its Ca2+-channel functions. Nat Neurosci. 2009;12(11):1415–23. Epub 2009/10/13. doi: 10.1038/nn.2417 ; PubMed Central PMCID: PMC2996863. 19820706 PMC2996863

[42] Theis AK, Rozsa B, Katona G, Schmitz D, Johenning FW. Voltage Gated Calcium Channel Activation by Backpropagating Action Potentials Downregulates NMDAR Function. Front Cell Neurosci. 2018;12:109. Epub 2018/05/15. doi: 10.3389/fncel.2018.00109 ; PubMed Central PMCID: PMC5932410. 29755321 PMC5932410

[43] Heck J, Palmeira Do Amaral AC, Weissbach S, El Khallouqi A, Bikbaev A, Heine M. More than a pore: How voltage-gated calcium channels act on different levels of neuronal communication regulation. Channels (Austin). 2021;15(1):322–38. Epub 2021/06/11. doi: 10.1080/19336950.2021.1900024 ; PubMed Central PMCID: PMC8205089. 34107849 PMC8205089

[44] Bakker MJ, Tijssen MA, van der Meer JN, Koelman JH, Boer F. Increased whole-body auditory startle reflex and autonomic reactivity in children with anxiety disorders. J Psychiatry Neurosci. 2009;34(4):314–22. Epub 2009/07/02. ; PubMed Central PMCID: PMC2702449. 19568483 PMC2702449

[45] Ferron L, Kadurin I, Dolphin AC. Proteolytic maturation of alpha2delta controls the probability of synaptic vesicular release. Elife. 2018;7. Epub 2018/06/20. doi: 10.7554/eLife.37507 ; PubMed Central PMCID: PMC6029843. 29916807 PMC6029843

[46] Guryev V, Koudijs MJ, Berezikov E, Johnson SL, Plasterk RH, van Eeden FJ, et al. Genetic variation in the zebrafish. Genome Res. 2006;16(4):491–7. Epub 2006/03/15. doi: 10.1101/gr.4791006 ; PubMed Central PMCID: PMC1457036. 16533913 PMC1457036

[47] Audira G, Siregar P, Strungaru SA, Huang JC, Hsiao CD. Which Zebrafish Strains Are More Suitable to Perform Behavioral Studies? A Comprehensive Comparison by Phenomic Approach. Biology (Basel). 2020;9(8). Epub 2020/08/06. doi: 10.3390/biology9080200 ; PubMed Central PMCID: PMC7465594. 32752218 PMC7465594

[48] McDiarmid TA, Belmadani M, Liang J, Meili F, Mathews EA, Mullen GP, et al. Systematic phenomics analysis of autism-associated genes reveals parallel networks underlying reversible impairments in habituation. Proc Natl Acad Sci U S A. 2020;117(1):656–67. Epub 2019/11/23. doi: 10.1073/pnas.1912049116 ; PubMed Central PMCID: PMC6968627. 31754030 PMC6968627

[49] Bleckmann H, Zelick R. Lateral line system of fish. Integr Zool. 2009;4(1):13–25. Epub 2009/03/01. doi: 10.1111/j.1749-4877.2008.00131.x . 21392273

[50] Orefice LL, Zimmerman AL, Chirila AM, Sleboda SJ, Head JP, Ginty DD. Peripheral Mechanosensory Neuron Dysfunction Underlies Tactile and Behavioral Deficits in Mouse Models of ASDs. Cell. 2016;166(2):299–313. Epub 2016/06/14. doi: 10.1016/j.cell.2016.05.033 ; PubMed Central PMCID: PMC5567792. 27293187 PMC5567792

[51] Alassaf M, Daykin EC, Mathiaparanam J, Wolman MA. Pregnancy-associated plasma protein-aa supports hair cell survival by regulating mitochondrial function. Elife. 2019;8. Epub 2019/06/18. doi: 10.7554/eLife.47061 ; PubMed Central PMCID: PMC6594750. 31205004 PMC6594750

[52] Westerfield M. The zebrafish book: a guide for the laboratory use of zebrafish (Brachydanio rerio). Eugene, OR: M. Westerfield; 1993.

[53] Kimmel CB, Ballard WW, Kimmel SR, Ullmann B, Schilling TF. Stages of embryonic development of the zebrafish. Dev Dyn. 1995;203(3):253–310. Epub 1995/07/01. doi: 10.1002/aja.1002030302 . 8589427

[54] Gyda M, Wolman M, Lorent K, Granato M. The tumor suppressor gene retinoblastoma-1 is required for retinotectal development and visual function in zebrafish. PLoS Genet. 2012;8(11):e1003106. Epub 2012/12/05. doi: 10.1371/journal.pgen.1003106 ; PubMed Central PMCID: PMC3510048. 23209449 PMC3510048

[55] Neff MM, Turk E, Kalishman M. Web-based primer design for single nucleotide polymorphism analysis. Trends Genet. 2002;18(12):613–5. Epub 2002/11/26. doi: 10.1016/s0168-9525(02)02820-2 . 12446140

[56] Untergasser A, Nijveen H, Rao X, Bisseling T, Geurts R, Leunissen JA. Primer3Plus, an enhanced web interface to Primer3. Nucleic Acids Res. 2007;35(Web Server issue):W71-4. Epub 2007/05/09. doi: 10.1093/nar/gkm306 ; PubMed Central PMCID: PMC1933133. 17485472 PMC1933133

