# Supplementary figures and images for: cacna2d3, a voltage-gated calcium channel subunit, functions in vertebrate habituation learning and the startle sensitivity threshold
Source: PLoS One. 2022 Jul 14;17(7):e0270903. doi: 10.1371/journal.pone.0270903 (PMC9282658; doi:10.1371/journal.pone.0270903)

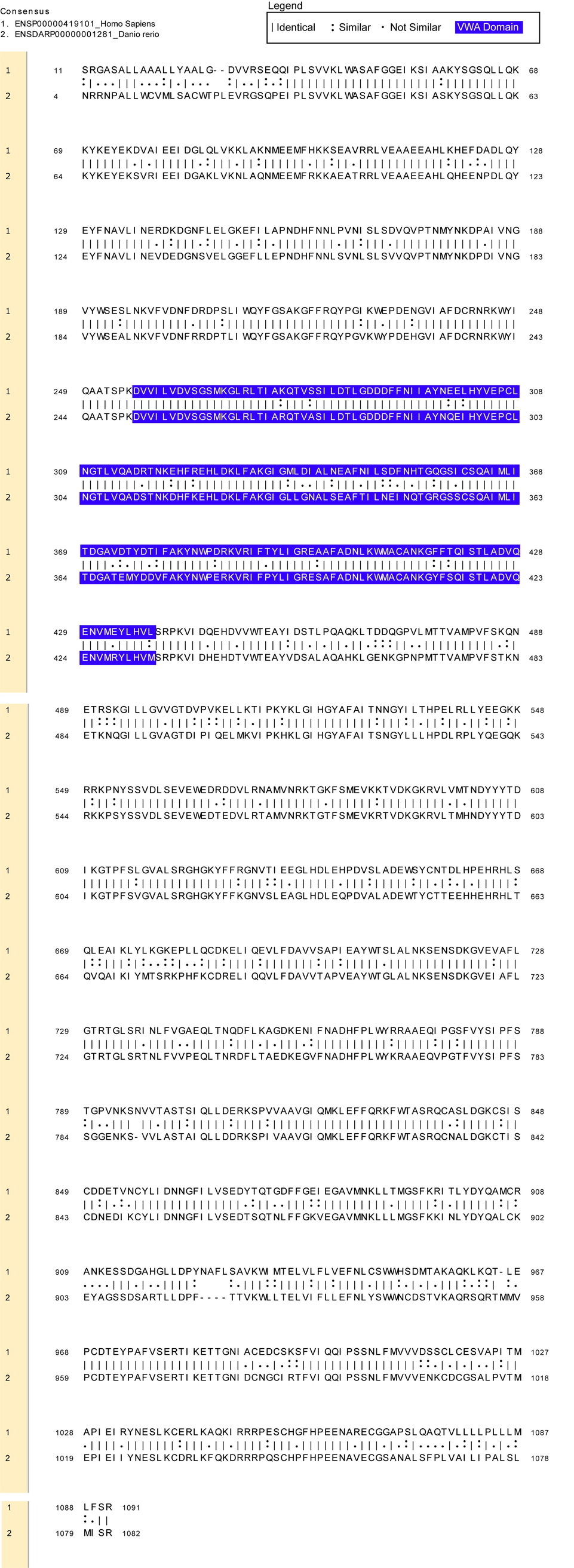

Supplement: S1 Fig — The alignment between human CACNA2D3 and zebrafish Cacna2d3 protein sequence was generated using the local alignment algorithm (Smith-Waterman) in SnapGene software. Human CANCA2D3 and zebrafish Cacna2d3 proteins share 76.5% amino acid identity and 87.6% similarity along the length of the protein. In the Von Willebrand Factor A (VWA) functional domain, the proteins share 89.4% amino acid identity and 93.4% similarity. Human CACNA2D3 is designated by row 1 and zebrafish Cacna2d3 is designated by row 2. VWA domain designated by blue box. | = identical amino acid;: = similar amino acid;. = not similar amino acid. (TIF) [file pone.0270903.s001.tif]

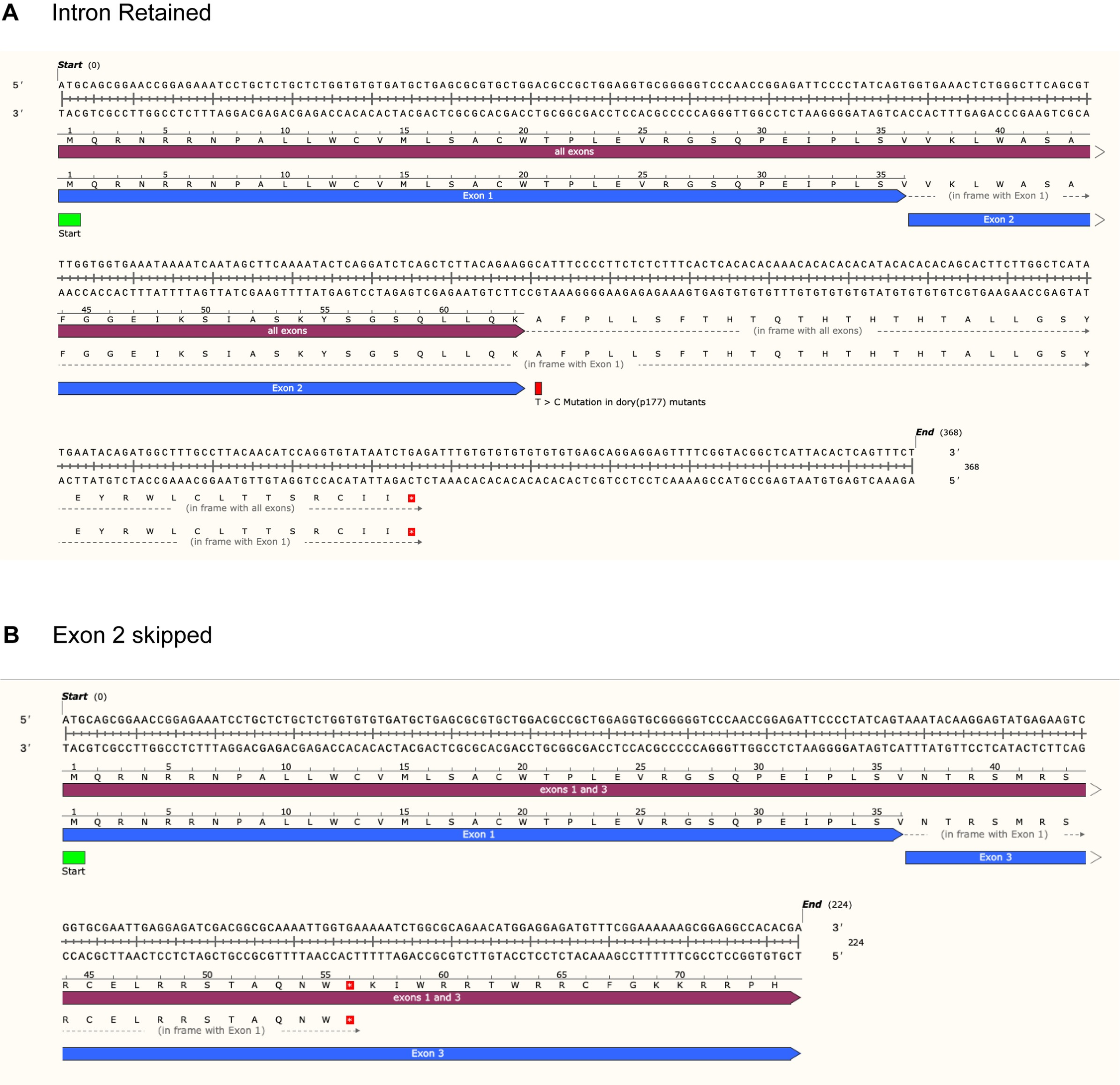

Supplement: S2 Fig — Predicted amino acid sequences encoded by doryp177 if the mutation causes retention of intron 2–3 (A) and if it causes skipping of exon 2 (B). (TIF) [file pone.0270903.s002.tif]
